# The Association of Cardioprotective Medications with Pneumonia-Related Outcomes

**DOI:** 10.1371/journal.pone.0085797

**Published:** 2014-01-28

**Authors:** Albert Wu, Chester Good, John R. Downs, Michael J. Fine, Mary Jo V. Pugh, Antonio Anzueto, Eric M. Mortensen

**Affiliations:** 1 Medical Service, South Texas Veterans Health Care System, San Antonio, Texas, United States of America; 2 Department of Medicine, University of Texas Health Science Center at San Antonio, San Antonio, Texas, United States of America; 3 Departments of Epidemiology and Biostatistics, University of Texas Health Science Center at San Antonio, San Antonio, Texas, United States of America; 4 VA Center for Health Equity Research and Promotion, VA Pittsburgh Healthcare System, Pittsburgh, Pennsylvania, United States of America; 5 Department of Medicine, University of Pittsburgh, Pittsburgh, Pennsylvania, United States of America; 6 Medical Service, VA North Texas Health Care System, Dallas, Texas, United States of America; 7 Departments of Internal Medicine and Clinical Sciences, University of Texas Southwestern Medical Center, Dallas, Texas, United States of America; College of Pharmacy, University of Florida, United States of America

## Abstract

**Introduction:**

Little research has examined whether cardiovascular medications, other than statins, are associated with improved outcomes after pneumonia. Our aim was to examine the association between the use of beta-blockers, statins, angiotensin converting enzyme (ACE) inhibitors, and angiotensin II receptor blockers (ARBs) with pneumonia-related outcomes.

**Materials and Methods:**

We conducted a retrospective population-based study on male patients ≥65 years of age hospitalized with pneumonia and who did not have pre-existing cardiac disease. Our primary analyses were multilevel regression models that examined the association between cardiovascular medication classes and either mortality or cardiovascular events.

**Results:**

Our cohort included 21,985 patients: 22% died within 90 days of admission, and 22% had a cardiac event within 90 days. The cardiovascular medications studied that were associated with decreased 90-day mortality included: statins (OR 0.70, 95% CI 0.63–0.77), ACE inhibitors (OR 0.82, 95% CI 0.74–0.91), and ARBs (OR 0.58, 95% CI 0.44–0.77). However, none of the medications were significantly associated with decreased cardiovascular events.

**Discussion:**

While statins, ACE inhibitors, and ARBs, were associated with decreased mortality, there was no significant association with decreased CV events. These results indicate that this decreased mortality is unlikely due to their potential cardioprotective effects.

## Introduction

Pneumonia affects 4 million people annually and is the eighth leading cause of death in the United States [1]. In 2007, in the United States there were 1.1 million hospitalizations due to pneumonia [2]. The number of patients admitted to hospitals for pneumonia is increasing, which may be due to an increase in an aging population as well as an increase in the number of co-morbid conditions [3].

Several studies have indicated that pneumonia may be associated with increased risk of heart disease, the leading cause of death in the United States [4]–[6]. These studies suggest that patients with concurrent pneumonia and cardiac events have significantly higher mortality than patients who only had pneumonia [4], [7]. Recent studies have shown that the use of statins and/or angiotensin-converting enzyme (ACE) inhibitors prior to admission is associated with decreased mortality in patients hospitalized with pneumonia [8]–[10]. It is unclear, however, whether this is due to cardioprotective effects or non-cardiovascular beneficial effects of these medications. In addition, while research has linked pneumonia and cardiovascular events, it is unclear whether use of cardioprotective medications, other than statins, are associated with improved clinical outcomes, such as mortality or cardiac events, for patients with pneumonia.

The aim of our study was to examine the association between the use of cardioprotective medications (e.g., beta-blockers, statins, ACE inhibitors, and ARBs) and 90-day mortality, and hospital admission due to cardiovascular events within 90-days, for male patients ≥65 years of age hospitalized with pneumonia using the extensive data of the Department of Veterans Affairs administrative databases. We hypothesized that in patients hospitalized with pneumonia, use of these cardiovascular medications would be associated with lower 90-day mortality and fewer cardiovascular events within 90-day of hospitalization.

## Materials and Methods

For this retrospective population-based study we used the administrative databases of the Department of Veterans Affairs (VA) Health Care System. These databases are the repositories of clinical data from all of the VA hospitals and outpatient clinics [11]. The Institutional Review Boards of the University of Texas Health Science Center at San Antonio and VA North Texas Health Care System approved this study. A waiver of informed consent was obtained from both ethics boards, as this was a retrospective study.

### Inclusion/Exclusion Criteria

Subjects included in this study met all of the following criteria:

- Age 65 or older on the date of admission.
- Had at least one outpatient clinic visit in the year preceding the index admission.
- Received at least one active and filled outpatient medication within 90-days of admission.
- Were hospitalized during fiscal years 2002–2007 (Oct 2001–Sep 2007) with a validated discharge diagnosis of pneumonia/influenza- either a primary ICD-9 codes 480.0–483.99 or 485–487 [12] or a secondary discharge diagnosis of pneumonia with a primary diagnosis of respiratory failure (ICD-9 code 518.81) or sepsis (ICD-9 code 038.xx) [12].
- Received at least one dose of antimicrobial therapy within the first 48 hours of admission.
- Did not have a pre-existing history of cardiac disease as defined previously [5]. We excluded those with a prior history of coronary artery disease, congestive heart failure, and/or arrhythmias as for subjects with these cardiac conditions we were unable to determine if a subsequent diagnosis was due to a new cardiac event or that the treating physicians felt that the pre-existing cardiac disease complicated the hospital stay.

We excluded women due to the small number who meet the inclusion criteria (n = 438). If a subject was admitted more than once for pneumonia during the study period, only the first hospitalization was included.

### Data

We used demographic, utilization, and comorbidity data from the National Patient Care Database, pharmacy data from the VA Decision Support System National Data Extracts (DSS NDE) and Pharmacy Benefits Management (PBM), and vital status information from VA's Vital Status file, which incorporates data from veterans' death benefits claims, inpatient deaths, Medicare Vital Status files, and the Social Security Administration death master file. Encrypted patient identifiers linked the information across these databases.

We obtained demographic information (age, sex, race, marital status) from inpatient and outpatient data. Race categories included white, black, Hispanic, and other/unknown. To infer active smoking and/or tobacco cessation attempts, we identified ICD-9 codes for tobacco use (305.1, V15.82), smoking cessation clinic use, and/or use of medications for the treatment of nicotine dependence (Zyban, nicotine replacement, or varenicline). Priority groups include (a) at least 50% disabled by a military service-connected condition (priority group 1), (b) up to 40% service-connected disability or special wartime cohorts such as recent Afghanistan or Iraqi veterans (priority groups 2–6), and (c) higher income patients with no service connected injuries (priority groups 7–8). We used VA priority status as a proxy for socioeconomic status.

We also obtained information on comorbid conditions from inpatient and outpatient administrative data. Alcohol abuse was defined by ICD-9 codes 291, 303, 305.0, and illicit drug use by ICD-9 codes 292, 304, 305 excluding 305.0-.1. We used the Charlson comorbidity index to quantify levels of preexisting comorbidity [13], adapted for administrative databases, using ICD-9 codes for 19 comorbid conditions from prior outpatient and inpatient encounters.

Pharmacy data were obtained from the PBM databases as well as from the DSS NDEs. Subjects were considered a current user of a given medication if they had enough pills to last until the date of hospitalization assuming an 80% compliance rate. To further adjust for potential confounding, a count of unique drugs in each of the following classes per patient was calculated for drugs prescribed within 90-days of presentation: other cardiac (excluding statins, ACE inhibitors, ARBs, and beta-blockers), pulmonary and diabetic medications. In addition, a dichotomized variable was created to identify those with intravenous or oral corticosteroid use within 90-days prior to hospitalization.

We also assessed for intensive care unit admission, receipt of invasive mechanical ventilation, and/or need for vasopressors, all within 48 hours of the index admission.

### Definition of exposure

Medications were classified as: statins (atorvastatin, cerivastatin, fluvastatin, lovastatin, pravastatin, and simvastatin); ACE inhibitors (benazepril, captopril, enalapril, fosinopril, lisinopril, moexipril, quinapril, and ramipril); ARBs (candesartan, irbesartan, valsartan, losartan, telmisartan, eprosartan, and olmesartan); or beta-blockers (metoprolol, atenolol, carvedilol, propranolol, bisoprolol, labetalol, nadolol, pindolol, and timolol). We created dichotomous variables to identify use of statins, ACE inhibitors, ARBs, or beta-blockers, which we defined as a filled prescription for the medication of interest within 90-days of presentation, with a sufficient supply to overlap the date of admission, assuming 80% compliance.

### Outcomes

Our primary outcomes were 90-day all-cause mortality or any cardiovascular event (e.g. myocardial infarction, congestive heart failure, and/or cardiac arrhythmia) within 90 days after admission. New cardiac events were identified after the date of admission using inpatient ICD-9 criteria [5]. Mortality was determined using the VA Vital Status File, which has been demonstrated to be as accurate as the “gold standard” National Death Index.

### Statistical Analyses

Bivariate statistics were used to test the association of sociodemographic and clinical characteristics with outcomes of 90-day mortality or cardiovascular events within 90-days. Categorical variables were analyzed using the chi-square test and continuous variables were analyzed using Student's t-test. To analyze time to mortality or cardiovascular events for patients by cardiovascular medications, we used a Kaplan-Meier graph to display the failure functions. Statistical significance was assessed using the log-rank test.

Next we created two generalized linear mixed-effect regression models (“multi-level model”) to examine the association of the cardiovascular medications on either 90-day mortality or cardiovascular events within 90-days with the patient's hospital as a random effect. Other factors included in the models included demographics, intensive care unit admission, need for mechanical ventilation and/or vasopressors, prior comorbid conditions, and other outpatient medications.

Comparisons were considered statistically significant for two-tailed p-values≤0.05. All analyses were performed using STATA 13 (StataCorp LP, College Station, Texas).

## Results

The cohort included 21,985 patients with a mean age of 74.6 years and 52% were married (Table 1). Whites comprised 80% of the cohort, 13% were black and 7% were of Hispanic ethnicity. In this cohort the most common comorbid conditions were chronic obstructive pulmonary disease (COPD) 48%, uncomplicated diabetes 26%, non-metastatic cancer 25% and peripheral vascular disease 12%. Mortality at 90 days was 22%, and 22% had a new cardiac event within 90 days of hospitalization.

**Table 1:** Demographic and Clinical Characteristics of Patients Hospitalized with Pneumonia (N = 21,985).

Variables	N (%)
**Demographics**	
Age at admission, mean (SD)	74.6 (6.8)
White	17,592 (80.0)
Black	2,880 (13.1)
Hispanic	1,553 (7.0)
Married	11,616 (52.8)
**Characteristics of hospitalization**	
ICU admission	2,791 (12.7)
Vasopressor Use	911 (4.1)
Mechanical ventilation	1,377 (6.3)
VA Priority group 1	3,744 (17.1)
VA Priority group 2–6	16,177 (73.7)
VA Priority group 7–8	2,036 (9.3)
**Comorbid conditions**	
Tobacco use/cessation	8,388 (38.2)
Alcohol abuse	1,092 (4.9)
Drug abuse	244 (1.1)
Peripheral vascular disease	2,536 (11.5)
Chronic obstructive pulmonary disease	10,628 (48.3)
Rheumatologic disease	604 (2.8)
Cirrhosis	199 (0.9)
Dementia	1,201 (5.5)
Diabetes	5,766 (26.2)
Diabetes with complication	1,478 (6.7)
Liver disease	92 (0.4)
Paraplegia	350 (1.6)
Renal disease	1,698 (7.7)
Cancer	5,479 (24.9)
Cancer with metastasis	924 (4.2)
AIDS	69 (0.3)
Charlson score, mean (SD)	2.3 (2.2)
**Medications**	
Pulmonary medications, mean (SD)	1.2 (1.9)
Diabetes medications, mean (SD)	0.3 (0.6)
Cardiac medications, mean (SD)	1.2 (1.4)
Corticosteroids, mean (SD)	0.3 (0.7)
Beta-blockers	4,855 (22.1)
Statins	5,301 (24.1)
ACE inhibitors	4,121 (19.7)
ARBs	535 (2.5)
**Outcomes**	
90-day mortality	4,873 (22.2)
Any cardiovascular event within 90 days	4,808 (21.9)
Myocardial Infarction within 90 days	1,009 (4.6)
CHF within 90 days	3,862 (17.6)
Arrhythmia within 90 days	3,984 (18.1)

**Figure 1:**
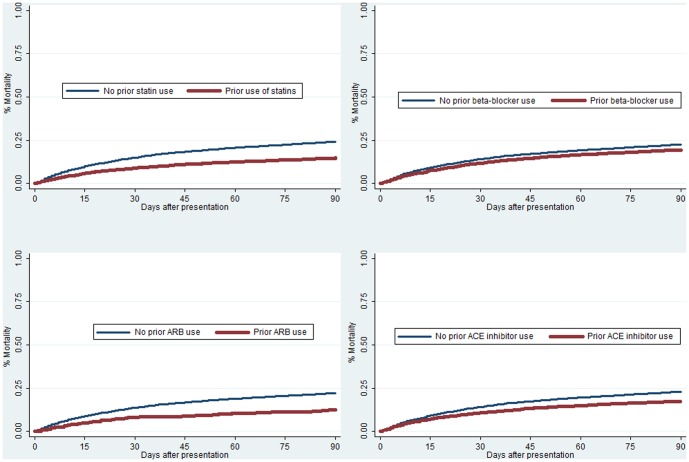
Mortality within 90-days by Prior Cardiovascular Medications Received (all p<0.001).

**Figure 2:**
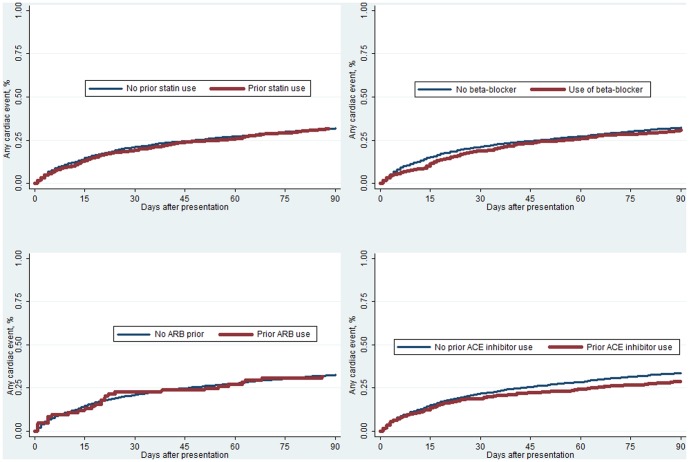
Percentage of the Cohort With Incident Cardiovascular Events by Prior Cardiovascular Medication Use (only significant association was with ACE inhibitors p = 0.03).

Figure 1 demonstrates time to death over time by prior cardiovascular medication use. Patients who had prior use of statins, beta-blockers, ARBs, and ACE inhibitors, all had significantly lower rates of mortality as compared to non-users (all p-values<0.0001.) Figure 2 demonstrates time to event for new cardiovascular events over time by prior cardiovascular medication use. Patients who received ACE inhibitors had significantly lower rates of new cardiovascular events as compared to non-users (p = 0.03.) There was no significant association between statin, ARB, or beta-blocker use (all p-values>0.5) and cardiovascular events.

To examine the impact of the medications of interest on our outcomes we performed two multilevel regression analyses. The first examined the impact of these medications on 90-day mortality after adjusting for other potential confounders (Table 2). Cardiac medications associated with decreased 90-day mortality included statins (OR 0.70, 95% CI 0.63–0.77), ACE inhibitors (OR 0.82, 95% CI 0.74–0.91), and ARBs (OR 0.58, 95% CI 0.44–0.77). Beta- blockers (OR 0.98, 95% CI 0.89–1.09) had no significant association with 90-day mortality. For the outcome of any cardiac event within 90-days none of the potential protective medications were significantly associated with decreased events (Table 3).

**Table 2:** Results of Multivariable Regression of Model for Outcome of 90-day Mortality.

Variable	Odds Ratio	95% CI	P-Value
Beta Blockers	0.98	0.89–1.09	0.96
Statins	0.70	0.63–0.77	<0.001
ACE inhibitors	0.82	0.74–0.90	<0.001
ARBs	0.58	0.44–0.77	<0.001
Age at admission	1.04	1.04, 1.05	<0.001
Black	0.98	0.88–1.09	0.72
Hispanic	0.84	0.71–1.04	0.12
Married	0.96	0.89–1.03	0.20
ICU	2.47	2.21–2.77	<0.001
Vasopressor Use	2.89	2.42–3.46	<0.001
Mechanical Ventilation	2.14	1.82–2.52	<0.001
Priority groups 2–6	1.15	1.05–1.27	0.003
Priority groups 7–8	1.05	0.91–1.21	0.49
Smoking	0.77	0.71–0.84	<0.001
Alcohol Abuse	0.92	0.78–1.10	0.29
Drug abuse	0.64	0.43–0.95	0.03
Peripheral vascular disease	1.18	1.06–1.32	0.002
Chronic obstructive pulmonary disease	0.98	0.89–1.06	0.56
Rheumatologic disease	0.97	0.77–1.20	0.77
Cirrhosis	1.74	1.20–2.55	0.004
Dementia	1.47	1.28–1.70	<0.001
Diabetes	1.12	1.01–1.26	0.04
Diabetes with complication	0.97	0.8–1.13	0.67
Liver disease	1.56	0.91–2.68	0.11
Paraplegia	1.08	0.82–1.42	0.58
Renal disease	1.22	1.08–1.40	0.002
Cancer	1.63	1.50–1.77	<0.001
Cancer with metastasis	3.49	3.00–3.10	<0.001
AIDS	1.03	0.51–2.01	0.92
Other cardiac medications*	0.93	0.90–0.96	<0.001
Pulmonary medications*	0.99	0.97–1.01	0.53
Diabetes medications*	0.93	0.96–1.01	0.09
Corticosteroids*	1.06	1.01–1.12	0.03

* Per each additional prescription.

**Table 3:** Results of Multivariable Regression of Model for Outcome of Incident Cardiovascular Event within 90 days.

Variable	Odds Ratio	95% CI	P-Value
Beta Blockers	1.01	0.93–1.13	0.64
Statins	1.10	0.99–1.20	0.06
ACE inhibitors	1.02	0.93–1.12	0.56
ARBs	1.013	0.82–1.28	0.81
Age at admission	1.03	1.03–1.04	<0.001
Black	0.74	0.65–0.83	<0.001
Hispanic	0.75	0.62–0.91	0.004
Married	0.93	0.87–1.0	0.051
ICU	3.11	2.78–3.50	<0.001
Vasopressor Use	0.95	0.78–1.14	0.57
Mechanical Ventilation	1.10	0.93–1.30	0.25
Priority groups 2–6	0.96	0.87–1.05	0.003
Priority groups 7–8	1.04	0.91–1.20	0.49
Tobacco use/cessation	0.99	0.91–1.07	0.73
Alcohol abuse	0.98	0.82–1.16	0.78
Drug abuse	1.21	0.86–1.70	0.27
Peripheral vascular disease	1.09	0.98–1.21	0.11
Chronic obstructive pulmonary disease	0.99	0.91–1.08	0.85
Rheumatologic disease	1.07	0.86–1.33	0.52
Cirrhosis	0.99	0.64–1.53	0.97
Dementia	0.65	0.55–0.77	<0.001
Diabetes	1.10	0.98–1.22	0.09
Diabetes with complication	1.02	0.87–1.19	0.82
Liver disease	0.93	0.49–1.74	0.81
Paraplegia	0.45	0.31–0.64	<0.001
Renal disease	1.05	0.92–1.19	0.48
Cancer	0.86	0.79–0.94	0.001
Cancer with metastasis	0.72	0.58–0.94	0.003
AIDS	0.87	0.43–1.75	0.69
Other cardiac medications*	1.10	1.07–1.13	<0.001
Pulmonary medications*	1.03	1.01–1.05	0.006
Diabetes medications*	1.04	0.96–1.12	0.32
Corticosteroids*	0.94	0.89–0.99	0.04

* Per each additional prescription.

## Discussion

Patients hospitalized for pneumonia are at high risk for cardiovascular events within 90 days of hospitalization [5], [14], [15] and patients with concurrent pneumonia and cardiac events have significantly higher mortality than patients who only had pneumonia [16]. Our study sought to examine whether cardioprotective medications are associated with both lower mortality and cardiovascular events in order to better define if the previously demonstrated lower pneumonia-related mortality associated with these medication are due to cardioprotective effects. While we found that statins, ACE inhibitors, and ARBs were associated with lower mortality, there was no significant association between these medications and lower cardiovascular events. Thus, our data suggests that beneficial effects of statin, ACE inhibitors, and ARBs on mortality in patients admitted with pneumonia may not be due to prevention of future cardiac events, but other mechanisms.

There is considerable literature demonstrating a significant number of cardiac events in patients with pneumonia [4]–[6], [14], [15]. One possible explanation is that an acute increase in inflammatory cytokines [17], [18] leads to instability of previously established atherosclerotic plaques [19]. Another possible mechanisms include mismatches between oxygen supply and demand may also cause increased cardiovascular events for patients with pneumonia [4], [6]. Finally, many of the bacteria that cause pneumonia have demonstrated the ability to directly infect cardiomyocytes, and cause conduction and contractility dysfunction [20]–[23]. For example, the most common cause of community-acquired pneumonia, *Streptococcus pneumoniae*, has recently been implicated in causing decreased cardiac contractility through immune-mediated uptake of bacterial cell wall antigen into cardiomyocytes [24].

There are several potential reasons that these medications may be protective against pneumonia-related mortality besides the demonstrated cardioprotective effects of statins, ACE inhibitors, and ARBs [10]. Both statins and ACE inhibitors have been demonstrated to blunt systemic inflammatory responses, which are also triggered in pneumonia [25], [26]. Statins also affect coagulation, cellular apoptosis, inflammatory cell signaling, leukocyte-endothelial cell adhesion, nitric oxide balance, and have anti-bacterial effects [27]. ACE inhibitors have also been demonstrated to have protective pulmonary effects [28], and two recent studies demonstrated that a genetic polymorphism associated with increased activity of the renin-angiotensin system is associated with increased incidence of, or higher mortality from, acute respiratory distress syndrome [29], [30].

There were several limitations to this study. Due to the small number of female patients available we were unable to examine if similar results would be found for women. Also the VA population is generally more medically complex and socioeconomically deprived than the general population so it is unclear how generalizable these results are [31]. We were also unable to examine whether the cause of pneumonia (bacterial or viral) influenced our results due to the study design. In addition, death certificate cause of death information was not available however prior studies have demonstrated that this information is frequently unreliable [32], [33]. Another limitation was reliance on ICD-9 diagnosis of cardiovascular events rather than clinical information, which particularly may impact the diagnosis of congestive heart failure. Not infrequently there is clinical confusion about whether patients have congestive heart failure, pneumonia, or both. We are unable to determine the extent to which these conditions may have been improperly differentiated. However due to our definition of pneumonia we are confident that the treating physicians believed that pneumonia was present. Also, we did not have clinical data, e.g., cardiac enzymes, B-type natriuretic peptide levels, radiography and echocardiogram results, available to confirm the diagnosis of cardiovascular events. Finally, we found it necessary to exclude those with pre-existing cardiac diagnoses from the sample due to difficulties in determining if subsequent discharge diagnoses represent new events or complicating factors during the hospitalization.

In conclusion, cardiovascular medications such as statins, ACE inhibitors, and ARBs, were associated with decreased mortality in patients hospitalized with pneumonia but not cardiovascular events. Additional research, especially prospective cohort and randomized trials, are needed to further examine the potential mechanism(s) of these associations and to determine if starting these medications at the time of diagnosis may be beneficial. For patients who are already taking these medications they should be continued unless there is direct contraindication (e.g., hypotension in a patient on ACE inhibitor or ARB).
